# Multidisciplinary management and nursing care for pediatric patients with febrile infection-related epilepsy syndrome in the acute phase: A case series

**DOI:** 10.1097/MD.0000000000049835

**Published:** 2026-07-17

**Authors:** Zi Lin, Tengjiao Zhang, Li Chen

**Affiliations:** a Department of Critical Care Medicine, Wuhan Children’s Hospital (Wuhan Maternal and Child Health Hospital), Tongji Medical College, Huazhong University of Science and Technology, Wuhan, Hubei Province, China.

**Keywords:** febrile infection-related epilepsy syndrome, intensive care nursing, ketogenic diet, multidisciplinary treatment, pediatric care, respiratory support

## Abstract

**Rationale:**

This case series discusses the nursing care and clinical outcomes of 3 pediatric patients with febrile infection-related epilepsy syndrome (FIRES) and acute-phase complications, focusing on critical care interventions such as seizure management, respiratory support, and nutritional therapy.

**Patient concerns:**

Three male patients, aged 10, 5, and 7, presented with altered consciousness, seizures, and recurrent fever. Diagnostic tests, including magnetic resonance imaging, electroencephalogram (EEG), and cerebrospinal fluid analysis, revealed abnormal EEG findings, suspected meningeal inflammation, and cerebrospinal fluid abnormalities.

**Diagnoses:**

All patients were diagnosed with FIRES, accompanied by acute neurological deterioration, supported by EEG and magnetic resonance imaging.

**Interventions:**

All patients required intensive care unit mechanical ventilation. Two underwent tracheostomy during prolonged ventilator dependence, whereas 1 was extubated to low-flow nasal oxygen after approximately 14 days without tracheostomy. Seizure management included anticonvulsants and a ketogenic diet, with individualized adjustments according to clinical response. Multidisciplinary care involved specialists in neurology, respiratory medicine, and rehabilitation.

**Outcomes:**

Short-term outcomes differed. One tracheostomized patient was later decannulated and discharged clinically improved, with recovery of communication, oral intake, and independent ambulation. The non-tracheostomized patient remained off invasive ventilation but continued inpatient neurologic and nutritional management, while the other tracheostomized patient still required ventilatory and neurologic management in the latest available record.

**Lessons:**

Coordinated multidisciplinary nursing, early nutritional planning, and structured monitoring may help organize acute-phase FIRES care while supporting individualized respiratory, seizure, and complication management.

## 1. Introduction

Febrile infection-related epilepsy syndrome (FIRES) is an exceptionally rare and often fatal condition that primarily affects previously healthy children. Its clinical presentation is marked by an abrupt onset of febrile infection accompanied by refractory epilepsy, which can swiftly progress to status epilepticus (SE).^[1–3]^ Despite its rarity, the profound physiological and psychological consequences of FIRES on affected children highlight the critical importance of early recognition and coordinated intervention, which may support timely clinical management and reduce preventable complications. The exact pathophysiology of FIRES remains incompletely elucidated, but existing studies suggest that inflammatory responses, immune-mediated mechanisms, and abnormal neuronal discharge are pivotal in its development.^[4–6]^ The interplay between infection and seizures may induce pathological changes through alterations in brain electrophysiological activity, immune system activation, and neuronal dysfunction. In the initial stages, affected children often present with high fever, intermittent seizures, altered consciousness, and even coma. The disease can persist for several weeks, with rapid progression, treatment challenges, and a high likelihood of various complications.^[7–9]^ Moreover, as a form of acute and prolonged SE, FIRES is characterized by distinct clinical features. Due to the extended duration of the disease and frequent seizure episodes, affected children often require intensive antiepileptic therapy. In addition to controlling seizures, treatment must address various factors, including airway management, temperature regulation, and nutritional support, necessitating a complex and multifaceted therapeutic approach.^[10–13]^

Nursing care during the acute phase of FIRES presents substantial challenges. Patients frequently experience uncontrollable, recurrent seizures, and as the condition progresses, they may develop altered consciousness, respiratory insufficiency, and other clinical manifestations that exacerbate disease severity.^[14]^ Consequently, nursing interventions during the acute phase must not only support seizure control but also continuously monitor vital signs, prevent complications, and ensure timely adjustment of treatment strategies. As FIRES involves multi-organ pathophysiological changes, children are at risk of not only cerebral injury but also life-threatening complications, including respiratory failure, infections, and malnutrition. Nursing staff must therefore be adept at applying a multidisciplinary collaborative approach, offering personalized, multilayered care. Comprehensive and systematic nursing interventions are important for supporting acute-phase management and may help reduce the risk of complications.^[15,16]^ Essential nursing measures for these patients include, but are not limited to, respiratory management, temperature regulation, seizure control, nutritional support, and complication prevention.^[17]^ Respiratory management is critical, as many children require mechanical ventilation due to seizures, sedative medications, or altered consciousness, with some even requiring tracheostomy. Given the frequent occurrence of hyperthermia and the limitations of pharmacological treatments, controlled cooling may be used to reduce harmful temperature fluctuations under close monitoring. Seizure control involves the judicious use of pharmacological agents and ketogenic diets (KDs), both integral to managing refractory epilepsy. In addition, nutritional support and the prevention of complications are vital components of acute-phase care that may influence short-term stability and recovery.^[18]^

Multidisciplinary team (MDT) collaboration is essential in the acute-phase care of FIRES patients. By integrating specialists from neurology, respiratory medicine, intensive care, rehabilitation, and nutrition, a cohesive and efficient team may improve the precision and timeliness of treatment while supporting individualized care plans for each patient.^[19]^ For example, neurologists are responsible for managing and adjusting antiepileptic medications, respiratory specialists oversee respiratory support and optimize ventilatory assistance, and nutritionists guide the design and adjustment of KD plans, with other specialists ensuring holistic care. During MDT implementation, regular case discussions facilitate timely modifications to treatment protocols and nursing strategies based on the evolving condition of the patient. Intensive care unit (ICU) nurses play a critical bedside role in comprehensive monitoring, neurologic observation, early recognition of complications, and precise execution of nursing interventions.^[20]^ Nurses also provide emotional support and facilitate communication with families, assisting both the child and their family in navigating this challenging period. The strength of MDT lies in the integration of specialized expertise across disciplines, enabling the formulation of evidence-based, rational treatment and care strategies. This collaborative approach may support timely treatment adjustment and recovery-oriented care. Although FIRES has received increasing clinical attention, research on acute-phase nursing care for these patients remains limited.

In comparison with existing nursing protocols for the management of refractory status epilepticus (RSE) and similar neurological conditions, our multidisciplinary care approach for FIRES is characterized by the integration of specialized interventions, including seizure monitoring, respiratory support, and early nutritional planning for KD therapy. Published guidance for SE and reviews of FIRES support coordinated acute management; however, detailed nursing protocols for pediatric FIRES remain scarce. Our case series presents a nursing model that highlights the early and coordinated implementation of these interventions, which may be beneficial for organizing acute-phase care. This comparison may help contextualize the nursing strategies used in this case series against broader acute-care principles. This article summarizes the acute-phase nursing experiences of 3 FIRES patients admitted to our hospital between August and December 2023, with the goal of providing guidance for clinical practice.

## 2. Case report

### 2.1. Ethics approval and consent to participate

This study was reviewed and approved by the Ethics Committee of Wuhan Children’s Hospital (approval number: 2022R063-E01). Written informed consent was obtained from the parents or legal guardians of all patients for participation and publication of this case series.

### 2.2. General information

Three male pediatric patients were admitted, all presenting with altered levels of consciousness and complaints of intermittent seizures accompanied by recurrent fever. Importantly, all patients were previously healthy children with no notable medical history.

Patient 1, a 10-year-and-4-month-old boy, was transferred to our hospital on August 3rd after undergoing intubation for airway management. Upon admission, he was in a sedated state with a body temperature of 36.6°C, and both the Kernig and Babinski signs were negative. Preadmission evaluations revealed abnormal electroencephalogram (EEG) findings, characterized by bilateral spike-and-slow wave discharges. Magnetic resonance imaging of the brain suggested possible meningeal inflammation, while computed tomography scans of the head, chest, and heart revealed no significant abnormalities. The serum concentration of valproate was measured at 16.42 μg/mL. Cerebrospinal fluid analysis before transfer indicated a white blood cell count of 14 × 10^6^/L (approximately 14 cells/μL), with 14% neutrophils and 86% lymphocytes. Biochemical analysis of the cerebrospinal fluid revealed sodium at 130.3 mmol/L, protein at 394 mg/L, and glucose at 5.23 mmol/L; C-reactive protein was <0.5 mg/L.

Patient 2, a 5-year-and-9-month-old boy, had a maximum temperature of 40.0°C prior to admission, with fever peaks occurring 3 times daily. Following 2 days of treatment at a local hospital, his fever showed improvement. However, 2 days prior to admission, he experienced a seizure during sleep, characterized by upward gaze, cyanosis of the lips, salivation, and limb rigidity, lasting approximately 5 minutes before subsiding. After this episode, he fell asleep and did not exhibit a fever. He was urgently taken to a local hospital, where he experienced another seizure with similar manifestations. After treatment with diazepam, his symptoms were alleviated. Subsequently, he was transferred to our hospital on September 8th, where he experienced another seizure, presenting as left upper limb rigidity lasting around 10 seconds.

Patient 3, a 7-year-and-2-month-old boy, had a maximum temperature of 39.3°C before admission and reported 3 episodes of vomiting after meals prior to seeking care at another hospital, where he was treated with Arbidol and Yinhuang oral solution. He presented with lethargy, drowsiness, and decreased appetite. Around noon on the day of admission, he exhibited upward gaze, cyanosis of the lips, foaming at the mouth, jaw clenching, bilateral lower limb rigidity, urinary incontinence, and altered consciousness, lasting approximately 4 hours. After receiving antipyretics and phenobarbital for seizure control, he continued to exhibit facial twitching and did not regain consciousness, prompting his transfer to our hospital on September 28th. Upon admission, his temperature was recorded at 37.6°C, and bilateral lung auscultation revealed slight wheezing, with a suspiciously positive Babinski sign and negative Kernig sign. One hour after admission, he experienced another seizure episode, characterized by upward gaze, salivation, and limb twitching, lasting a few seconds before subsiding, but seizures recurred.

### 2.3. Treatment and outcomes

All 3 critically ill pediatric patients were admitted to the pediatric intensive care unit (PICU) and placed under 24-hour multi-lead dynamic EEG monitoring. Upon admission, comprehensive assessments were completed, including routine blood tests, biochemical profiles, inflammatory markers, neuroimaging, and cardiopulmonary function assessments. All patients received endotracheal intubation and invasive mechanical ventilation during the acute PICU course. Two patients underwent tracheostomy during prolonged ventilator dependence. Patient 1 was later successfully weaned from mechanical ventilation, decannulated on November 1, and discharged clinically improved on November 5; Patient 2 remained on tracheostomy-assisted ventilation in the latest available record. Patient 3 did not undergo tracheostomy and was extubated to low-flow nasal oxygen on October 13, after approximately 14 days of ventilatory support. For seizure management, the patients received multidrug antiepileptic regimens, consistent with published new-onset refractory status epilepticus/FIRES consensus definitions and SE management principles.^[12,14,15]^ Blood gas analysis was performed every 2 hours to guide respiratory support adjustments.

Throughout the acute phase, an MDT consisting of pediatric intensivists, neurologists, immunologists, infectious disease specialists, and clinical nurses was established. The MDT conducted daily interdisciplinary rounds to dynamically adjust sedation strategies (e.g., midazolam), antiepileptic drugs (e.g., valproic acid, levetiracetam), and immunomodulatory therapies (e.g., intravenous immunoglobulin and cytokine-directed biologic therapy when clinically indicated). Notably, for patients exhibiting recurrent fever refractory to conventional antipyretics, we applied controlled cooling therapy using continuous cooling blankets, with core temperature measured every 4 hours.

The therapeutic approach followed a stepwise strategy that combined initial stabilization, etiology-oriented evaluation, and adjunctive therapies. Initial stabilization included mechanical ventilation, continuous EEG monitoring, sedation and analgesia when indicated, and antipyretic or controlled cooling therapy. Etiology-based treatment included antimicrobial stewardship and immunomodulation according to clinical evaluation. Adjunctive therapy included glucocorticoid boluses and KD initiation when clinically indicated for RSE.

Nursing interventions were implemented as structured bedside protocols rather than isolated tasks. These included 2-hour neurological assessments of consciousness, pupil size, and light reflexes, particularly during suspected seizure episodes; continuous observation for respiratory, hemodynamic, and temperature fluctuations; and supportive care aimed at preventing ventilator-associated pneumonia (VAP), deep vein thrombosis (DVT), pressure injuries, and nutritional intolerance. The nursing team also supported thermoregulation through scheduled temperature monitoring and controlled cooling application, and coordinated with physicians during respiratory mechanics optimization and spontaneous breathing trials when clinically appropriate.

The available follow-up period differed among patients. At the latest reviewed records, Patient 1 had been decannulated and discharged without respiratory support after recovery of communication, oral intake, and independent ambulation; Patient 3 had remained off invasive ventilation with stable spontaneous breathing while continuing neurologic and nutritional management; and Patient 2 still required tracheostomy-assisted ventilation with ongoing seizure and complication management. Therefore, these results are presented as short-term inpatient outcomes rather than uniform recovery.

## 3. Nursing

### 3.1. Implementation of multidisciplinary treatment models and personalized management plans

Multidisciplinary treatment (MDT) refers to a collaborative approach where experts from 2 or more distinct disciplines convene at a designated time and location to discuss the diagnosis and treatment decisions for patients with specific organ or system diseases. The MDT then collaboratively executes the agreed-upon decisions. In this study, we established an MDT team consisting of professionals from the PICU, neurology, respiratory medicine, interventional radiology, rehabilitation, radiology and imaging, and nutrition. The MDT members conducted in-depth case discussions, with PICU physicians presenting each patient’s condition, current treatment strategy, and clinical response. Subsequently, the team determined the next diagnostic and therapeutic steps, formulated personalized management plans, and created an institutional communication group for streamlined information sharing. ICU nurses assisted in developing daily treatment and nursing checklists, observing and recording patient conditions, performing risk assessments, establishing nursing plans, ensuring the implementation of therapeutic and nursing measures, and providing psychological support. Radiologists and imaging specialists reviewed imaging findings, rehabilitation specialists focused on early rehabilitation, and nutritionists evaluated and adjusted KD plans. Other specialists contributed to treatment decisions according to the child’s evolving clinical needs. During treatment, ICU nurses tracked the completion of tasks based on daily goal lists, documented unfinished items in the special transfer log, and ensured accurate bedside handovers. Disease progression, including consciousness status, seizure activity, temperature variation, psychological status, and complications, was reported during every shift through the institutional communication group. During morning rounds, the team reviewed the completion of goal lists from the previous day and updated each patient’s overall condition.

### 3.2. Respiratory management and improvement of respiratory function

During the acute phase of FIRES, respiratory function in children can be affected by frequent seizures, sedative medications, and impaired consciousness, necessitating mechanical ventilation. Published FIRES reviews indicate that severe acute episodes commonly require anesthetic treatment and mechanical ventilation, and tracheostomy may be required when ventilator dependence is prolonged.^[16]^ Key measures in respiratory management include: implementing bundled airway management to prevent VAP; administering effective sedation and analgesia to control seizures during mechanical ventilation; and providing structured care for patients with tracheostomy. Bundled airway management measures include: environmental control, maintaining a temperature of 22°C to 24°C and humidity between 50% and 60%, wiping down patient surfaces with disinfecting wet wipes twice daily, and periodically spraying chlorine-based disinfectants; elevating the head of the bed by 15° to 30°; conducting oral hygiene with chlorhexidine mouthwash, using a syringe and toothbrush in a specified order, 3 times daily; employing a closed suction system for airway suctioning, providing 2 minutes of pure oxygen before and after suctioning, maintaining suction pressure below 40 kPa (300 mm Hg), and limiting each suctioning episode to no more than 15 seconds; continuously monitoring cuff pressure with a pressure gauge and keeping it within 25 to 30 cmH_2_O, with remeasurement when necessary; regularly draining condensation from ventilator water collection cups to ensure proper equipment function; and promptly replacing contaminated or damaged ventilator tubing.

Limb movement and pupil changes were closely observed to detect possible seizure activity. In the acute phase, deep sedation and analgesia were used when clinically indicated; all 3 patients received midazolam for sedation and remifentanil for analgesia via continuous infusion pumps over 24 hours. As the patients stabilized, sedation strategies were gradually adjusted toward an eCASH-based approach, balancing adequate pain control, lighter sedation when feasible, and humanistic care. Pain levels were monitored with FLACC/NRS tools and maintained between 0 and 1 when possible. Sedation was adjusted from midazolam to dexmedetomidine according to clinical response, with target RASS scores between −2 and 0 and Ramsay scores between 2 and 4.

Tracheostomy care included: close monitoring for bleeding, subcutaneous emphysema around the incision, displacement, and tube obstruction; maintaining room temperature between 22°C and 24°C and humidity around 60%; covering the tracheostomy site with 2 to 4 layers of gauze and changing the dressing every 1 to 2 days or immediately when contaminated; replacing the outer cannula daily; ensuring that the tracheostomy tie allowed approximately 1 finger of space; using saline for airway humidification, instilling 1 to 2 mL every 15 to 30 minutes when needed; and performing thorough suctioning with a separate suction tube for each nursing session.

Respiratory outcomes varied across the 3 patients. Patient 1 underwent tracheostomy during the acute course, was successfully weaned from mechanical ventilation, and was later decannulated. Patient 2 underwent tracheostomy and still required tracheostomy-assisted ventilation in the latest available record reviewed. Patient 3 was successfully extubated to low-flow nasal oxygen after approximately 14 days of ventilatory support and did not undergo tracheostomy. When VAP was suspected, antibiotic therapy was adjusted according to clinical evaluation and microbiological evidence, and nursing measures such as frequent repositioning and postural drainage were intensified to prevent aspiration and respiratory complications.^[21]^

### 3.3. Comprehensive control of seizure activity and mitigation of seizure-induced brain injury

The acute phase of FIRES is primarily characterized by RSE, which can persist for 1 to 12 weeks and often responds poorly to conventional antiepileptic treatment. KD is an evidence-supported therapeutic option for refractory SE in FIRES patients and is also discussed in broader protocols for super-RSE.^[22–25]^ Early KD initiation may be considered as part of individualized seizure-management planning when clinically appropriate.

Three pediatric patients presented with frequent seizures both prior to and upon admission, despite receiving oral chloral hydrate for sedation. As seizures continued unabated, intravenous midazolam infusion was initiated, but this did not provide effective seizure control. Further treatment included continuous intravenous infusion of valproate, alongside oral antiepileptic agents such as oxcarbazepine, levetiracetam, topiramate, valproate (extended-release), perampanel, clonazepam, and lacosamide. During antiepileptic therapy, Patient 1 developed a widespread, dense papular rash on the torso, which resolved after discontinuing oxcarbazepine and applying fish liver oil ointment and a pruritus-relieving topical wash.

To meticulously monitor seizure events, a seizure recording form was developed at our institution. This form captured the seizure’s onset time, duration, manifestations, and the identity of the recorder. Nursing staff completed the records daily and handed them over during shift changes, enabling adjustments to the antiepileptic medication regimen based on the data collected. After initial pharmacological measures, seizure control remained unsatisfactory in the acute phase.

On the 5th day of hospitalization, Patient 1 began a KD (25 g of ketogenic powder Type I mixed with 140 mL water, 5 times daily), providing a total caloric intake of 625 kcal. On the 14th day, the patient entered a comatose state, likely due to insufficient caloric intake. After consultation with the nutritionist, the ketogenic regimen was revised to 7.5 g ketogenic powder Type I plus 200 mL ketogenic liquid milk, 5 times daily. By the 21st day, the patient’s weight had decreased, with wide fluctuations in blood ketones and low albumin levels. Nutritional status appeared poor, prompting further modification of the dietary plan to a 4:1 ratio ketogenic liquid milk (250 mL per dose, 5 times daily). By the 29th day, when KD was discontinued, seizure duration and clinical manifestations had improved compared with the earlier acute course.

Patient 2 commenced the KD on the 9th day of hospitalization (36 g ketogenic powder Type I mixed with 200 mL water, 5 times daily), with a total caloric intake of 1000 kcal. On the 9th day, diarrhea and abdominal bloating were observed, leading to modification of the diet to 30 g ketogenic powder Type I plus 11 g small peptides per dose, 5 times daily, yielding a total caloric intake of 1100 kcal. On the 13th day, the patient experienced a decrease in leg circumference, which was hypothesized to result from caloric insufficiency. The regimen was further adjusted to 35 g ketogenic powder Type I plus 4.6 g small peptides per dose, 5 times daily, with a total caloric intake of 1200 kcal. On the 21st day, when the KD was discontinued, the patient exhibited extended intervals between seizures, reduced frequency, and shorter seizure duration.

Patient 3 began a KD during hospitalization (40 g ketogenic powder Type I with water and 6 g small peptides per dose, 5 times daily), totaling 1300 kcal. After KD initiation, coffee-ground gastric aspirate or vomiting prompted a temporary reduction of the KD dose and intragastric thrombin administration. The regimen was later adjusted to 28.8 g ketogenic powder Type I, 17 g ketogenic powder Type III, and 6 g protein powder per dose, 5 times daily. By the time KD was discontinued, seizure manifestations had decreased compared with the acute phase.

Throughout the KD period, all 3 patients were also administered compound nutritional powder, ketogenic probiotics, and potassium citrate granules via nasogastric tube. During medication infusion, glucose was substituted with normal saline, and regular monitoring of blood glucose and ketone levels was conducted. Patient 1 experienced 3 episodes of blood ketones exceeding 5 mmol/L, which were promptly addressed with 30 mL of orange juice administered via nasogastric tube, followed by a dietary adjustment to restore normal levels.

Research indicates that KDs may provoke nutritional intolerance symptoms such as diarrhea, bloating, and vomiting.^[26]^ Patient 2 developed severe diarrhea and erythema with ulcerative bleeding in the buttocks. The nursing team responded by changing to breathable diapers, increasing the frequency of diaper changes, maintaining dryness, and applying local wound care and assisted drying measures to keep the ulcerated area clean and dry. After meticulous care over several days, the skin returned to normal.

### 3.4. Temperature management: preventing temperature-related fluctuations in disease progression

During the prodromal or acute course, all 3 patients had recurrent fever, with peaks around 39°C to 40°C and repeated temperature fluctuations despite conventional antipyretic treatment. The acute phase of FIRES is often accompanied by febrile responses, and elevated body temperature may provoke seizures.^[27]^ To prevent hyperthermia-induced seizures, anti-infective therapy was administered according to clinical evaluation and microbiological findings, and all patients underwent controlled cooling therapy using a cooling blanket to manage temperature. During controlled cooling therapy, the healthcare team vigilantly monitored the skin condition to prevent frostbite and regularly inspected the proper functioning of the cooling equipment.

Within 5 to 7 days of admission, the body temperature of all 3 patients was maintained below 38.5°C. For temperatures ranging from 37.5°C to 38.0°C, physical cooling methods such as open blankets and tepid sponge baths were employed. For temperatures between 38.0°C and 38.5°C, diclofenac sodium suppositories or oral ibuprofen were used as prescribed, resulting in gradual normalization of body temperature. Although controlled cooling therapy can reduce basal metabolic rate, it may also lead to complications such as hypothermia, arrhythmias, and hypotension^[28]^; therefore, continuous monitoring of vital signs, skin color, and limb temperature variations is essential. Both Patient 1 and Patient 2 developed hypokalemia, which was promptly treated with 10% potassium chloride orally, resulting in normalization. Patient 1 experienced hypotension, with mean arterial pressure dropping below 50 mm Hg, necessitating rapid administration of norepinephrine infusion as per the medical order, maintaining mean arterial pressure above 70 mm Hg.

### 3.5. Multidimensional prevention and precision treatment of deep venous thrombosis

Due to factors such as inflammatory responses, antiepileptic drug use, prolonged bed rest, mechanical ventilation, and controlled cooling therapy, critically ill pediatric patients with FIRES may be vulnerable to venous thromboembolism; pediatric hospitalized populations have shown an increasing VTE burden.^[29]^ DVT can prolong hospitalization and increase morbidity. In hospitalized medical populations, anticoagulant prophylaxis has been shown to reduce symptomatic VTE risk.^[30]^

At admission, all 3 patients underwent thrombosis risk assessment using an in-hospital pediatric venous thrombosis risk assessment sheet (Version 1.0), because all patients were aged ≤13 years. Adult tools such as the Padua Prediction Score^[31]^ and the Caprini risk assessment model^[32]^ were not used to classify these 3 children; they are noted here only as institutional tools for older medical patients or surgical patients. Low- and moderate-risk patients were reassessed every 7 days, and high-risk patients were reassessed every 3 days, with timely reevaluation after changes in risk factors. For low-risk patients, physical prophylactic measures were implemented, while moderate- and high-risk patients received pharmacological prophylaxis when clinically indicated.

Preventive care included infection control and early mobilization, as infections can trigger inflammatory responses, damage endothelial cells, and promote thrombus formation. Antimicrobial therapy was adjusted according to clinical evaluation, microbiological findings, and antimicrobial susceptibility results, while nursing care emphasized catheter-site observation, limb-circumference monitoring, and prompt escalation when swelling or perfusion changes were noted.

Early mobilization promotes circulation and reduces the risk of thrombosis. During bed rest, patients were assisted in repositioning every 2 hours, underwent passive ankle pump exercises, and had limb warmth maintained during controlled cooling therapy. For pharmacological prophylaxis in moderate- and high-risk patients, low-molecular-weight heparin (LMWH) was recommended for DVT prevention, with strict control of dosage and regular monitoring of coagulation function.

Patient 1 received subcutaneous LMWH prophylaxis during the ICU course when thrombosis risk was elevated. Patient 2 developed catheter-associated thrombosis involving the left lower limb; central venous catheterization was discontinued, LMWH anticoagulation was initiated, and serial ultrasound follow-up showed reduction and later recovery-phase change rather than an abrupt single-time-point resolution. Patient 3 was assessed as having moderate VTE risk, but prophylactic LMWH was deferred initially because of concern regarding intracranial bleeding. These individualized decisions were made jointly by the ICU, cardiovascular, interventional, and neurology teams.

### 3.6. Individualized psychological care

Due to hypoxic brain injury associated with SE, FIRES patients often exhibit altered consciousness, with uncontrolled seizures further complicating the recovery of awareness. To facilitate consciousness recovery, the nursing team provided a calm and comfortable environment and employed various sensory stimuli to promote awakening. Family members were encouraged to engage with patients through bi-weekly video visits, with recorded messages or music from loved ones played to maintain emotional connection. In addition, family members were given the opportunity for in-person visits to foster interaction and aid in the patient’s cognitive recovery.

## 4. Discussion

FIRES is an acute refractory epileptic encephalopathy that typically follows a febrile infection and may progress to prolonged RSE. Its pathophysiology remains incompletely understood, although immune activation and neuroinflammatory mechanisms have been proposed.^[1]^ The 3 children in this case series were previously healthy boys who developed fever, impaired consciousness, and recurrent seizures with limited early response to conventional antiepileptic treatment. Their clinical course was therefore consistent with recognized FIRES features. Diagnosis in such cases remains primarily clinical and requires careful exclusion of alternative infectious or structural causes, supported by magnetic resonance imaging and continuous EEG assessment.^[10,33]^

The acute-phase care described here was organized through an MDT model involving PICU clinicians and nurses, neurology, respiratory medicine, rehabilitation, interventional radiology, and nutrition. Evidence from broader critical care settings suggests that multidisciplinary ICU teams may be associated with improved outcomes,^[34]^ but in this small FIRES case series, the main value of MDT collaboration was practical: rapid reassessment, coordinated adjustment of antiepileptic and respiratory support, and consistent bedside execution of nursing plans. ICU nurses contributed to neurologic observation, seizure documentation, temperature monitoring, family communication, and early recognition of complications.

Respiratory management was central because all 3 patients required invasive mechanical ventilation during the acute PICU course. Published FIRES and new-onset refractory status epilepticus reports similarly describe the frequent need for anesthetic treatment, midazolam infusion, and ventilatory support during prolonged SE.^[13,16]^ Two patients underwent tracheostomy during prolonged ventilator dependence, but their subsequent courses differed: Patient 1 was successfully weaned from mechanical ventilation, decannulated, and discharged clinically improved, whereas Patient 2 still required tracheostomy-assisted ventilation in the latest available record. Patient 3 did not undergo tracheostomy and was extubated to low-flow nasal oxygen after approximately 14 days. These differences support individualized airway planning and close monitoring for VAP and airway complications.^[21]^

KD, thermoregulation, and thrombosis prevention required individualized nursing support rather than a uniform protocol. KD is recommended in pediatric dietary-therapy guidance for epilepsy and has been discussed as an option in refractory and super-RSE management.^[23–25]^ In our patients, KD use required repeated adjustment because of nutritional intolerance, weight change, ketone fluctuations, and gastrointestinal symptoms. Fever control was also important because hyperthermia may aggravate neurologic injury and provoke seizures; therefore, controlled cooling was used with close monitoring for hypothermia, hypotension, arrhythmia, skin injury, and electrolyte disturbance.^[35]^ Because prolonged bed rest, mechanical ventilation, infection, central venous access, and inflammation may increase venous thrombosis risk in critically ill children, DVT prevention and treatment were individualized according to risk assessment and bleeding concerns.^[29,30]^

This report has several limitations. It includes only 3 patients from a single center, and the available follow-up duration was not uniform across cases. The study had no control group, and the concurrent use of antiepileptic drugs, immunomodulatory therapies, respiratory support, and KD prevents attribution of clinical changes to any single intervention. Therefore, the findings should be interpreted as descriptive acute-phase nursing experience rather than evidence of treatment efficacy. Larger multicenter studies with standardized follow-up are needed to clarify which care components most strongly influence short- and long-term outcomes in pediatric FIRES.

## Acknowledgments

The authors thank the families of the patients for their cooperation and consent to participate in this study.

## Author contributions

**Methodology:** Zi Lin.

**Resources:** Zi Lin, Tengjiao Zhang, Li Chen.

**Data curation:** Tengjiao Zhang.

**Project administration:** Li Chen.

**Writing – original draft:** Zi Lin.

**Writing – review & editing:** Zi Lin.
